# Ghrelin Upregulates *Hoxb4* Gene Expression in Rat Bone Marrow Stromal Cells

**DOI:** 10.22074/cellj.2018.5164

**Published:** 2018-03-18

**Authors:** Alireza Abdanipour, Behnaz Shahsavandi, Mohsen Alipour, Hadi Feizi

**Affiliations:** 1 Department of Anatomical Sciences, Faculty of Medicine, Zanjan University of Medical Sciences, Zanjan, Iran; 2 Department of Physiology and Pharmacology, Faculty of Medicine, Zanjan University of Medical Sciences, Zanjan, Iran

**Keywords:** Bone Marrow Stromal Cells, Ghrelin, HOXB4, Rat

## Abstract

**Objective:**

Ghrelin is a peptide which has a proliferative and antiapoptotic effect in many cells including bone marrow stromal cells (BMSCs). Homeobox protein B4 (HOXB4) is a transcription factor involved in stem cell regeneration and survival. The aim of the study was to find out the effect of ghrelin on *Hoxb4* expression in BMSCs.

**Materials and Methods:**

In this experimental study, rat BMSCs were cultivated in Dulbecco’s Modified Eagle Medium (DMEM). Passage three BMSCs were treated with ghrelin 100 μM for 48 hours. Real-time polymerase chain reaction (PCR) was carried out from the untreated BMSCs (B), BMSCs treated with 125 µM H_2_O_2_ (BH), BMSCs treated with 100 µM ghrelin then 125 µM H_2_O_2_(BGH) and BMSCs treated with 100 µM ghrelin (BG) groups. For immunofluorescence, cells were incubated with an anti-HOXB4 monoclonal antibody. Primary antibodies were visualized using the Fluorescein isothiocyanate (FITC) method. All data are presented as mean ± SEM and P<0.05 was considered as statistical significant.

**Results:**

Hoxb4 expression significantly increased in the BG compared with BH and BGH groups. Furthermore, 100 µM ghrelin, increased the mean of HOXB4 positive immunoreactive cells compared to the BH group.

**Conclusion:**

Ghrelin probably enhances proliferation and viability of BMSCs through Hoxb4 upregulation. However, the signaling pathway and other biological outcomes of this effect should be elucidated in different stem cells.

## Introduction

Ghrelin is an endogenous peptide, mostly released from the fond ues of stomach, is well known as a growth hormone, secretagogue and a metabolism regulator (1). However, some other physiological roles for this peptide have been introduced by researchers during the last decade. Regarding to stem cells, it has been shown that ghrelin is involved in both proliferation and differentiation of various types of stem cells and also has a protective function. Ghrelin induces proliferation of hippocampal neural stem cells and this effect was eliminated by adding the peptide receptor antagonist in the cultured cells’ medium (2, 3). Ghrelin promotes human embryonic stem cell differentiation to cardiomyocytes (4, 5). Furthermore, ghrelin increases the regeneration of bone marrow stem cells (6). 

Bone marrow stromal cells (BMSCs) are a population of cells that structurally and physiologically support the hematopoietic cells (7). Moreover, these cells have stem cell characteristics leading to their differentiation into bone, cartilage, adipocytes, and hematopoietic supporting tissues (8). Furthermore, it has been noted that MSCs have important roles in immune regulation (9). Recently we have shown that addition of ghrelin to BMSCs culture medium increases their proliferation and also protects them against H_2_O_2_-induced apoptosis and thus increases their viability (10). 

Homeobox proteins are transcription factors which are involved in development (11). *In vivo* and in vitro studies emphasize that expression of *Hoxb4*, a member of the Homeobox proteins, expands stem cells especially hematopoietic stem cells (HSCs) (12-14). In addition, it has been shown that *Hoxb4* is involved in the inhibition of apoptotic cell death (15, 16). The aim of the present study was to find the effect of ghrelin on *Hoxb4* expression in BMSCs in order to reveal the probable mechanism of the proliferative and anti-apoptotic effect of this peptide in BMSCs.

## Materials and Methods

### Bone marrow stromal cell culture and drug treatments 

In this experimental study, all the procedures were carried out under approval from the Ethical Committee of Zanjan University of Medical Sciences (ZUMS.REC.1394.164). Rat BMSCs were expandedin Dulbecco’s Modified Eagle Medium (DMEM, Gibco, USA), supplemented with 20% fetal bovine serum (FBS, Gibco, USA), 100 U/ml penicillin, and 100 mg/ml streptomycin (Gibco, USA). Subsequently, cells were incubated at 37°C (5% CO_2_) in the 25 cm^2^ plastic flasks. The medium was refreshed every 2-3 days until cells became confluent. Cells were harvested with trypsin-EDTA and passaged up to three times. Ghrelin was freshly prepared to treat BMSCs. Passage-three BMSCs were cultured in 96-well plates (5000 cells/well) in DMEM medium supplemented with ghrelin (100 µM) for 48 hours (10).

### Real-time polymerase chain reaction

Real-time polymerase chain reaction (PCR) was carried out with RNA from the untreated BMSCs (B), BMSCs treated with 125 µM H_2_O_2_ (BH), BMSCs treated with 100 µM ghrelin then 125 µM H_2_O_2_ (BGH) and BMSCs treated with 100 µM gherelin (BG) groups. In all groups, 1,000 ng purified RNA from cultured cells was used to synthesize 20 µl cDNA, using Revert aid™ first strand cDNA synthesis kit (Fermentas, Germany) according to the manufacturer’s instructions. To quantify *Hoxb4* mRNA levels, cDNA (25 ng) was used. *GAPDH* primers were used as an internal control. All primers have been listed in Table 1. The PCR reaction was synthesized in a 12.5 µl volume (containing sense and anti-sense primers, cDNA, and Sybr green) and performed for 40 cycles using an Applied Biosystems thermal cycler. We used delta delta CT method (Pfaffl method) for analyzing relative changes in mRNA levels.

**Table 1:** Sequences of oligonucleotide primers


Name	Sequence ID	Primer sequences (5´→ 3´)

*HoxB4*	NM_001100787.1	F: GCGACCATTACCTCGACACT
		R: GTTACCGTGGCCAAAACACT
*GAPDH*	XM_017593963.1	F: CAAGGTCATCCATGACAACTTTG
		R: GTCCACCACCCTGTTGCTGTAG


### Immunostaining 

BMSCs were cultured on cover slips and fixed in 3% paraformaldehyde (Merck, Germany) for 20 minutes at RT, followed by a permeabilization step in 100% methanol (Merck, Germany) for 30 minutes at RT. For immunofluorescence, cells were incubated with anti-CD90 (for BMSCs) and anti-HOXB4 (for produced erythroid progenitor cells) monoclonal antibodies, followed by incubation with a fluorescein isothiocyanate (FITC)-conjugated rabbit anti-mouse antibody (Millipore). Nuclei were counterstained with DAPI. For indirect immunoperoxidase labeling, 100 µM ghrelintreated BMSCs (for 48 hours) were permeabilized with 0.4% Triton X-100 (Merck, Germany), followed by 10% fetal calf serum (FCS) for 60 minutes to block endogenous peroxidases. Then were incubated with antiCD90 and anti-HOXB4 antibodies overnight at 4°C. The FITC method was used for the visualization of primary antibodies. 

### Statistical analysis

In this study, SPSS15 software was used for statistical analysis. All data are presented as mean ± SEM. To compare multiple means in groups, one-way ANOVA followed by Tukey’s post hoc comparison was used. Values of P<0.05 were considered statistically significant.

## Results

### *Hoxb4* gene expressions evaluation 

**Fig.1:**
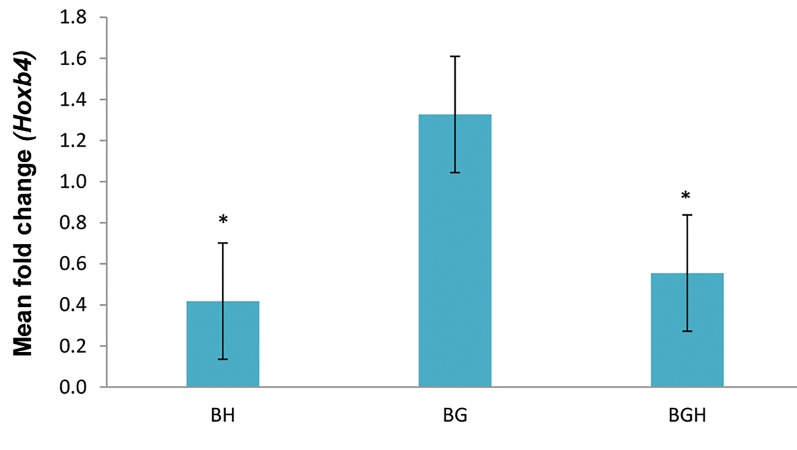
*Hoxb4* mRNA expression. Fold change ratio of Hoxb4 mRNA in BMSCs treated with 100 µM concentration of ghrelin for 48 hours for various groups. Real-time polymerase chain reaction (PCR) results have been presented as relative expression normalized to GAPDH mRNA amplification. Amplification of the Hoxb4 mRNA derived from the BMSCs treated with 125 µM H_2_O_2_ (BH), BMSCs treated with 100 µM ghrelin (BG) and BMSCs treated with 100 µM ghrelin then 125 µM H_2_O_2_ (BGH) groups showing increased levels of Hoxb4 mRNA after 100 µM ghrelin treatment. The bars indicate the mean ± SEM. *; P<0.05 (compared to the BG group) and BMSCs; Bone marrow stromal cells.

Increasing in *Hoxb4* mRNA transcription in BMSCs treated with 100 µM concentration of ghrelin for various groups (BH, BG and BGH) at 48 hours was confirmed through quantitative real-time reverse transcriptase PCR (RT-PCR). The results of the mRNA expression assessments have been shown in the (Fig .1). Our data showed that mRNA expressions of *Hoxb4* significantly increased when ghrelin was used (P<0.05). Also in the 100 µM ghrelin-treated group, mRNA expressions were significantly up-regulated compared to the BH group at 48 hours (P<0.05). The results demonstrated a significant increase of Hoxb4 mRNA levels in the BG group (1.32 ± 0.1) compared to the BH (0.41 ± 0.02) and BGH (0.55 ± 0.02) groups (P<0.05).

### HOXB4 protein production evaluation

**Fig.2:**
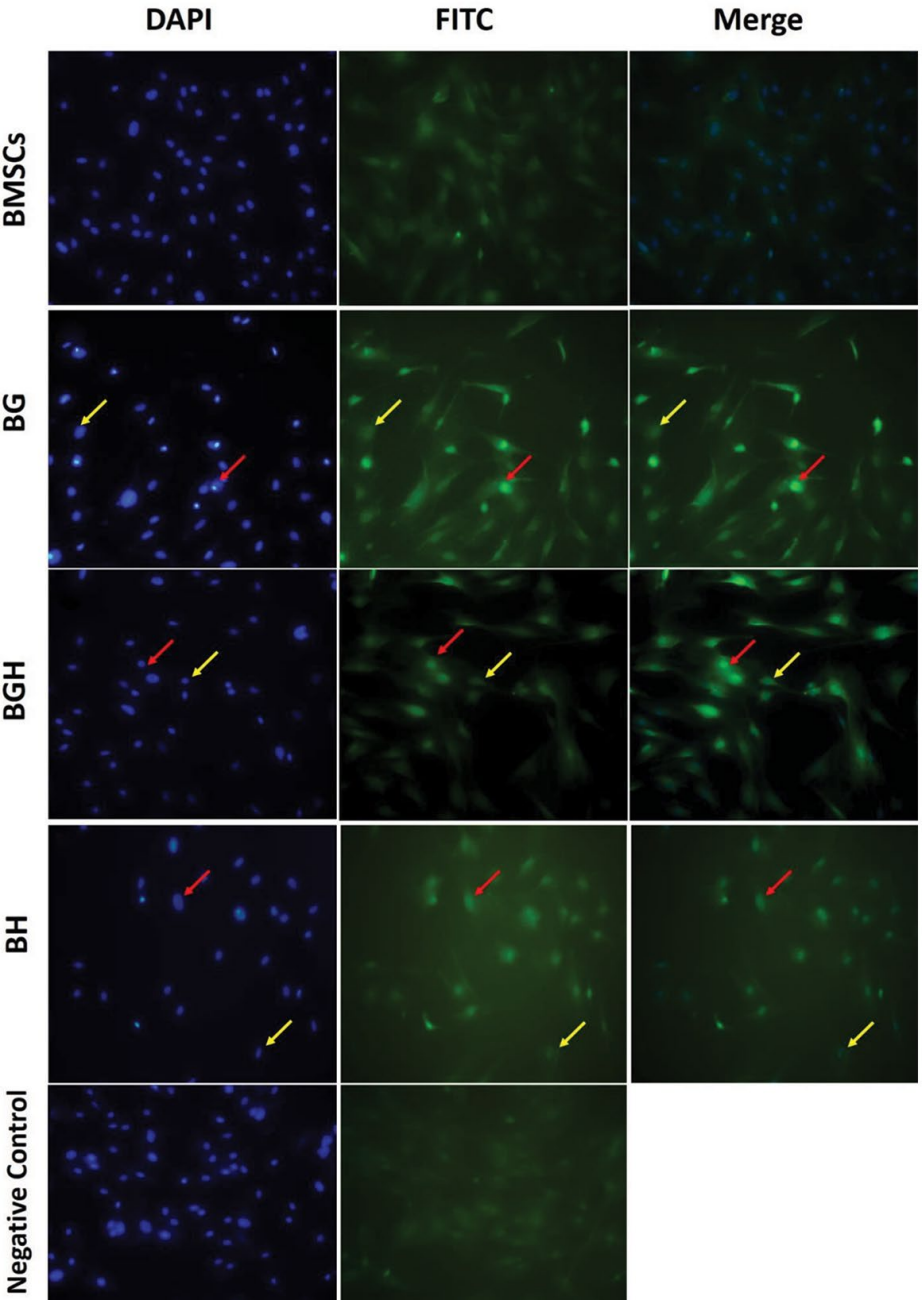
HOXB4 protein expression. Representative immunostaning photomicrographs showing HOXB4 immunoreactivity in the BMSCs treated with 125 μM H_2_O_2_ (BH), BMSCs treated with 100 μM ghrelin (BG) and BMSCs treated with 100 μM ghrelin then 125 μM H_2_O_2_ (BGH) groups after 48 hours of treatments. Red arrows indicate the immunopositive cells and yellow arrows indicate negative cells (magnification: ×200). BMSCs; Bone marrow stromal cells.

**Fig.3:**
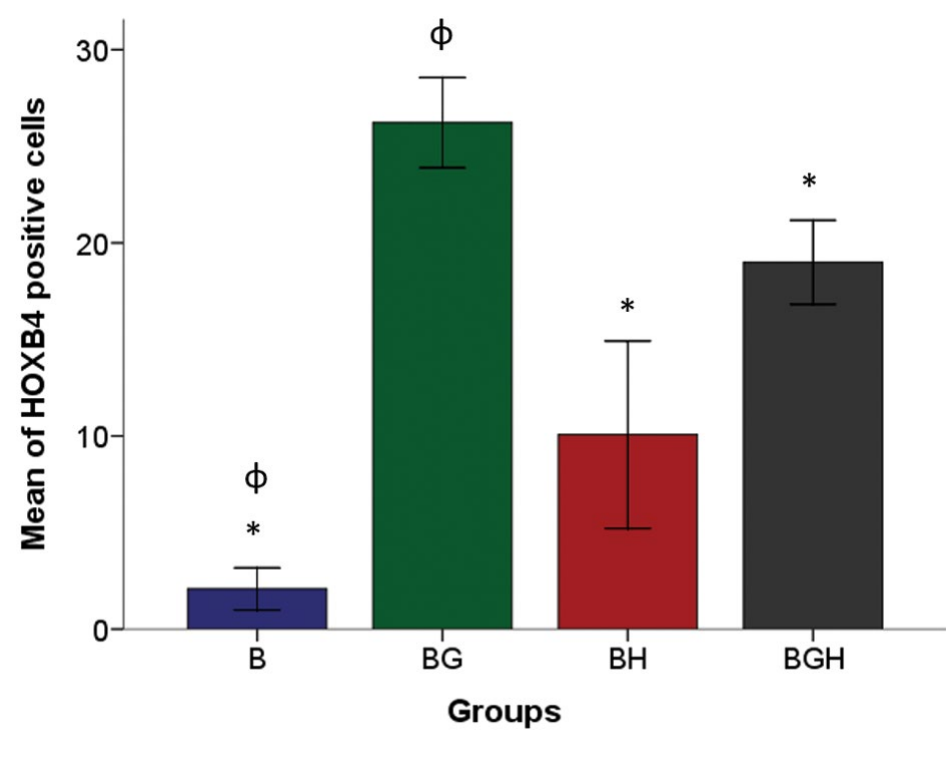
The mean percentage of the HOXB4 positive cells in the experimental groups. The bars indicate the mean ± SEM. *; Compared to the BMSCs treated with 100 µM ghrelin (BG) group and ɸ; compared to the BMSCs treated with 100 µM ghrelin then 125 µM H_2_O_2_(BGH) group and P<0.05. BMSCs; Bone marrow stromal cells.

 In the immunocytochemistry evaluation, we observed that BMSCs treated with 100 µM ghrelin were positively stained for HOXB4 (Fig .2). The mean of positive cells as shown in figure 3, were 2.08 ± 0.54, 26.22 ± 1.16, 10.06 ± 2.42 and 18.99 ± 1.08 for the B, BG, BH and BGH groups respectively. Ghrelin treatment significantly increased the positive cells in BGH compared to the BH group (P<0.05, Fig.3). 

## Discussion

The results of this study have shown, for the first time, that ghrelin upregulates the *Hoxb4* at both the mRNA and protein production levels in BMSCs. As mentioned before, ghrelin increased BMSC proliferation in our latest study (10). It has been clearified by Chung and colleagues that several transcription factors are involved in the proliferative effect of ghrelin (17). Now, we can add *Hoxb4* to the list of transcription factors which are influenced by ghrelin.

Ghrelin also increased the *Hox-B4* gene expression and protein production in H_2_O_2_-exposed BMSCs, but it was not significant. Earlier studies by Morel and Barouki (18) revealed that oxidative stress could lead to repression of various genes’ expression as including some transcription factors. Therefore, this may be the possible cause that H_2_O_2_-treated BMSCs did not represent significant elevated *Hoxb4* expression in response to ghrelin.

In this new study we have shown that ghrelin protects BMSCs from H_2_O_2_-induced apoptosis (10). Daniels et al. (15) reported that Hoxb4 overexpression in malignant B-cells makes them resistant to apoptosis. Furthermore, Park and his colleagues reported that overexpression of *Hoxb4* in Ba/F3 cells, diminished cell death through Fas protein stimulation (16). So, it is possible that the mentioned effect of ghrelin in protecting the BMSCs against H_2_O_2_ stress, to some extent, could be due to partial induction of *Hoxb4* expression. A couple of studies have demonstrated that most of the protective effects of ghrelin take place through the PI3-AKT and/or MAPK signaling pathways (19, 20). However, we did not investigate whether ghrelin increases *Hoxb4* gene expression through these pathways.

In our previous study, it was indicated that ghrelin induces severe polycythemia in the rats living in hypoxia (21). In a complementary study, the relation between ghrelin administration and erythropoietin production has been elucidated and we concluded that the polycythemic effect of ghrelin was not through erythropoietin upregulation (22). It is believed that BMSCs produce cytokines that support HSC function and are involved in the regulation of hematopoiesis (23, 24). Maybe ghrelin at least by means of regulating BMSCs could affect hematopoiesis and therefore lead to polycythemia.

A few studies have shown that *Hoxb4* possesses a regulatory role in the self-renewal of HSCs (13, 25). On the other hand, overexpression of *Hoxb4* in non-hematopoieticstem cells could differentiate them to hematopoietic fates. Lee et al. (14) have shown that overexpression of *Hoxb4* in embryonic stem cells (ESCs) using lentiviruses, increases their differentiation to HSCs. Later, Forrester and his colleagues demonstrated a paracrine effect for HOXB4 in which its forced expression in ESCs increased production of Frzband other growth factors such as fibroblast growth factor (FGF) and transforming growth factor (TGF) leading to their differentiation to HSCs (26, 27). We did not evaluate the hematopoietic biomarkers in BMSCs undergoing *Hoxb4* overexpression, as a result we cannot deduce whether or not these cells have been differentiated to hematopoietic cells. However, in our forthcoming study we are going to examine this phenomenon. So, if proved, ghrelin could be introduced as a new agent to improve both *in vitro* HSC harvest and therapeutic strategies for patients with hematopoietic disorders.

## Conclusion

Ghrelin upregulates the *Hoxb4* gene expression in rat BMSCs and this phenomenon may be involved in proliferative and antiapoptotic effects of ghrelin. However, the signaling pathways and the application of this outcome should be elucidated in different stem cells including hematopoietic stem cells.
